# The Relationship of Metabolic Syndrome With Disease Activity and the Functional Status in Patients With Rheumatoid Arthritis

**DOI:** 10.4021/jocmr1001w

**Published:** 2012-07-20

**Authors:** Mehmet Karakoc, Ibrahim Batmaz, Mustafa Akif Sariyildiz, Mehmet Tahtasiz, Remzi Cevik, Ebru Tekbas, Ismail Yildiz, Tahsin Celepkolu

**Affiliations:** a Department of Physical Medicine and Rehabilitation, Dicle University School of Medicine, Diyarbakir, Turkey; b Department of Cardiology, Dicle University School of Medicine, Diyarbakir, Turkey; c Department of Biostatistics, Dicle University School of Medicine, Diyarbakir, Turkey; d Department of Family Practice, Dicle University School of Medicine, Diyarbakir, Turkey

**Keywords:** Romatoid arthritis, Metabolic syndrome, Disease activity.

## Abstract

**Background:**

The aim of this study is to investigate the frequency of metabolic syndrome (MS) in patients with rheumatoid arthritis (RA) and to determine the relationship between the clinical and laboratory parameters of RA and the components of the metabolic syndrome (MS).

**Methods:**

Fifty-four patients with RA and 52 healthy individuals were enrolled in this study. The diagnosis of rheumatoid arthritis was based on the American College of Rheumatology criteria and the diagnosis of the metabolic syndrome was made according to the criteria set out in the American Heart Association/National Heart, Lung, and Blood Institute (AHA/NHLBI). The functional status and disease activity were also recorded in patients with RA.

**Results:**

MS was diagnosed in 42.6% of the patients with RA and in 9.6% of the healthy controls. The systolic and diastolic blood pressure values were observed to be significantly higher in the patients with RA in comparison to the controls. Also, the frequency of MS was higher in the inferior functional group in relation to the higher functional group. A positive correlation was observed between the DAS28 scores and hypertension in patients with RA.

**Conclusions:**

In this study, MS was more frequently detected in the patients with RA compared to the control group. Also, an inferior functional status in RA was also found to be associated with the presence of MS. Thus, the presence of MS in patients with RA may be associated with a higher cardiovascular risk.

## Introduction

Rheumatoid arthritis (RA) is a chronic inflammatory disorder of unknown etiology, characterized by systemic symptoms that particularly involve the joints and may lead to deformities during the course of the disease [1]. Besides the frequent extra-articular involvement, RA is also associated with cardiovascular disorders. Various studies have pointed to a higher mortality rate in RA where 35-50% of the mortality was attributed to cardiovascular disease (CVD). Still, cardiac involvement has also been observed without the clinical symptoms of heart disease [2-6].

Metabolic Syndrome (MS) is a definition encompassing the important risk factors (hypertension, obesity, diabetes mellitus and lipid profile disorders) for cardiovascular diseases. The criteria set out in International Diabetes Federation (IDF) [7] and the American Heart Association/National Heart, Lung, and Blood Institute (AHA/NHLBI) are utilized in the diagnosis of MS [8]. Recent studies have suggested that the increase in the cardiovascular risk in RA may be related to the increased frequency of MS [9-11]. Besides, the effects of the increase of the inflammatory response in RA on the development of MS and CVD are also well known [12-14].

The aim of our study was to determine the frequency of MS in RA and to study the relationship between the clinical and laboratory parameters of RA and MS in order to shed light to the influence of MS in the aetiology of CVD that develops in patients with RA.

## Materials and Methods

Fifty-four patients with RA diagnosed according to the classification criteria defined by the American College of Rheumatology (ACR) [15] and followed up in the Physical Medicine and Rehabilitation Clinic were enrolled in the study, along with 52 healthy individuals of matching age and sex groups who served as the control group. The control group consisted of individuals who had applied to our outpatient clinic with nonspecific back pain. The approval of the local ethics committee was obtained prior to the initiation of the study and all the individuals who participated were given information about the study before they were presented with consent forms to sign.

Patients with any kind of concurrent collagen tissue disorders or other inflammatory articular diseases, malignancies, diseases of the central nervous system, chronic kidney disease, chronic liver disease and thyroid diseases besides RA, and those who were pregnant were excluded from the study.

After the detailed patient histories were obtained, all the patients underwent detailed systemic examinations and locomotor system screenings. Weight and height values were recorded. The waist circumference was measured with a tape measure at the slimmest point of the waist below the lower costal margin while the patient was standing upright naked from the waist up. The systolic and diastolic blood pressure values were measured from the right arm in due manner between 9.00 and 12.00, after the patients rested for 10 minutes in sitting position. Any pain in the patients with RA was evaluated with the help of the 100 mm visual analogue scale.

Laboratory tests included the complete blood count (CBC), routine biochemistry tests and the lipid profile. The erythrocyte sedimentation rate (ESR) was measured by the Westergren method (mm/h) and the serum C reactive protein (CRP) levels were measured through nephelometry (mg/dl). Patients were advised to fast after 20.00 on the night before the day of the blood tests. The venous blood samples were tested without delay. Although there are three different criteria to describe the MS, the most commonly preferred criteria are those defined in the International Diabetes Federation (IDF) and the American Heart Association/National Heart, Lung, and Blood Institute (AHA/NHLBI) [7, 8]. We have also based our study on the AHA/NHLBI criteria and diagnosed those patients with MS who tested positive for 3 or more out of the following 5 risk factors: Waist circumference (female > 88 cm, male > 102 cm), triglycerides ≥ 150 mg/dl, HDL cholesterol (female < 50 mg/dl, male < 40 mg/dl), blood pressure (≥ 130/85 mmHg) and fasting blood glucose (≥ 100 mg/dl).

The general health evaluations were carried out according to the Health Assessment Questionnaire (HAQ). The reliability and validity of the Turkish version of the HAQ have been studied by Kucukdeveci *et al* [16].

The functional status was assessed according to the ACR functional classification [17]. Patients who tested positive for MS were divided into two groups according to their functional status, with stages 1 and 2 defining higher functional status and stage 3 and 4 describing lower functional status.

In patients with RA, disease activity was evaluated with the help of the DAS-28 (Disease Activity Score) calculation, which is based on the number of the swollen and tender joints, the ESR and the visual analogue scale where the patient evaluates his/her own condition globally. The DAS-28 results were classified as follows: higher disease activity > 5.1; 5.1 - 3.2: medium disease activity; and lover disease activity ≤ 3.2 [18].

The statistical analyses were performed using the SPSS 16.0 software package. The compliance of the variables to the normal distribution was assessed through the Kolmogorov-Smirnov test. Inter-group differences were evaluated using the Mann-Whitney U-test. While the categorical and ratio-proportional variables (odds ratio) were assessed through the Chi-square test, the inter-variable relationships were evaluated with the help of Spearman’s correlation test. Statistical significance was based on a value of p < 0.05.

## Results

Both the RA and the control groups were similar in terms of age distribution (p > 0.05). The RF, ESR, CRP, leukocyte count, platelet count, globulin and total cholesterol values were higher in the patient group (Table 1).

**Table 1:** Demographic Features and Laboratory Findings of the RA and Control Groups

	Rheumatoid Arthritis	Controls	p
Age	49.76 ± 11.15	47.05 ± 9.75	0.188
Sex (M/F)	47/7	43/9	0.530
BMI	29.4	27.9	0.325
Duration of RA (years)	7.6		
RF (IU/ml)	114.37 ± 171.27	12.23 ± 3.88	0.001
CRP (mg/dl)	2.53 ± 3.78	0.60 ± 1.36	0.001
ESR (mm/h)	33.57 ± 22.91	13.81 ± 10.89	0.000
Leukocytes	8.95 ± 68.90	6.36 ± 0.44	0.000
Platelets	299.26 ± 86.78	240.85 ± 77.84	0.000
Albumin	3.68 ± 0.42	4.17 ± 0.34	0.000
Globulin	3.48 ± 0.57	3.17 ± 0.32	0.004
Total cholesterol (mg/dl)	190.70 ± 48.28	170.79 ± 31.38	0.014
LDL cholesterol (mg/dl)	111.21 ± 37.88	106.20 ± 30.17	0.454

F: Female; M: Male; BMI: Body mass index, RF: Rheumatoid Factor, CRP: C-Reactive Protein, LDL: Low Density Lipoprotein, ESR: Erythrocyte Sedimentation Rate.

While MS was detected in 23 patients (42.6%) out of the 54 in the RA group, it was observed in 5 (9.6%) among the 52 patients in the control group. The prevalence of MS among the patients with RA was significantly higher (p < 0.001). All of the MS parameters, except for the fasting blood sugar level, were higher in the RA group in comparison to the control group. However, only the difference in the systolic and diastolic blood pressure levels were statistically significant (p = 0.001, p = 0.011, respectively) (Table 2).

**Table 2:** The Ratio of MS and the Mean MS Parameters in the RA and Control Groups

	Romatoid Arthritis	Controls	p
MS (%)	23 (42.6%)	5 (9.6%)	0.000
Systolic blood pressure (mmHg)	122.59 ± 17.58	108.85 ± 24.04	0.001
Diastolic blood pressure (mmHg)	76.85 ± 11.91	70.48 ± 13.44	0.011
HDL cholesterol (mg/dl)	50.61 ± 12.27	48.40 ± 9.84	0.308
Triglycerides (mg/dl)	154.22 ± 105.22	135.62 ± 103.17	0.36
Fasting blood glucose (mg/dl)	99.11 ± 19.22	101.13 ± 21.32	0.608
Waist circumference (cm)	92.13 ± 17.04	91.73 ± 15.60	0.9

HDL: High Density Lipoprotein, MS: Metabolic Syndrome

The patients with RA were divided into two groups according to the presence of MS. No statistically significant difference was observed between the groups in terms of the CRP, RF, Anti-CCP, leukocyte count, ESR, disease duration, DAS-28 and VAS score mean values (p > 0.05). The mean age in the MS-positive RA group was higher than the MS-negative RA patients (p = 0.0001) (Table 3).

**Table 3:** The Demographic and Laboratory Parameters of the MS (+/-) RA Patients

	MS positive	MS negative	p-value
Age (years)	55.70 ± 9.55	45.35 ± 10.28	< 0.0001
CRP (mg/dl)	2.61 ± 4.28	2.49 ± 3.43	0.906
RF (IU/dl)	58.36 ± 55.77	154.38 ± 211.88	0.055
Anti-CCP (U/dl)	389.49 ± 396.42	322.43 ± 352.88	0.571
Leukocytes	9.12 ± 2.56	8.46 ± 1.99	0.07
ESR (mm/h)	31.39 ± 21.07	35.19 ± 24.40	0.552
Disease duration	10.74 ± 10.73	6.76 ± 4.97	0.49
DAS-28	3.68 ± 1.41	3.88 ± 1.50	0.627
VAS (cm)	3.91 ± 2.09	3.50 ± 2.28	0.499

RA: Rheumatoid Arthritis, MS: Metabolic Syndrome, RF: Rheumatoid Factor, CRP: C-Reactive Protein, ESR: Erythrocyte Sedimentation Rate, Anti-CCP: Anti-Cyclic Citrullinated Peptide, DAS-28: Disease Activity Score, VAS: Visual Analogue Scale

While a positive correlation was observed between the MS parameter of systolic blood pressure, and the age and DAS-28 scores; there was a negative correlation with the sedimentation rate. A positive correlation of the diastolic blood pressure was also detected with the age and the DAS 28 scores. The waist circumference and age were also positively correlated, while the HDL and RF were negatively correlated (Table 4).

**Table 4:** Correlation Between the Clinical and Laboratory Parameters and the MS Components in Patients With RA

	Systolic blood pressure	Diastolic blood pressure	Waist circumference	Glucose	HDL	Triglycerides
Age (years)	0.440*	0.410*	0.550**	0.130	-0.008	0.120
DAS 28	0.330*	0.350*	-0.110	0.110	-0.230	0.029
Disease duration (years)	0.090	0.210	-0.045	0.150	-0.220	0.250
HAQ	0.170	0.240	0.200	0.140	-0.230	0.200
ESR (mm/h)	-0.290*	-0.130	-0.150	-0.076	-0.054	-0.094
CRP (mg/dl)	-0.210	-0.049	-0.190	0.150	-0.150	-0.022
RF (IU/dl)	-0.130	-0.130	0.002	-0.037	0.310*	-0.058
Anti-CCP (U/dl)	-0.021	-0.036	0.230	-0.081	-0.100	0.044

RA: Rheumatoid Arthritis, MS: Metabolic Syndrome, RF: Rheumatoid Factor, CRP: C-Reactive Protein, ESR: Erythrocyte Sedimentation Rate, Anti-CCP: Anti-Cyclic Citrullinated Peptide, DAS-28: Disease Activity Score, VAS: Visual Analogue Scale, HDL: High Density Lipoprotein, LDL: Low Density Lipoprotein, HAQ: Health Assessment Questionnaire. *Statistically significant (p > 0.05) relationship with Spearman’s correlation test. **Statistically significant (p > 0.01) relationship with Spearman’s correlation test.

It has also been evaluated if the functional status is a risk factor for MS: There were 41 (75.9%) patients in the higher functional (stages 1 - 2) group and 13 patients (24.1%) in the lower functional (stages 3 - 4) group. Where 14 (34.1%) patients were MS (+) in the higher functional group, 9 patients (69.2%) were MS (+) in the lower functional group. Therefore, the risk for being positive for MS in the lower functional group was significantly higher than the good functional group (OR = 4.34, CI: 1.13 - 16.62; p = 0.032).

## Discussion

In this study, we have found the prevalence of MS significantly higher in patients with RA compared to the healthy controls. Also, all the parameters of MS, except for the fasting blood glucose values, were higher in the patients with MS. However, only the difference in the systolic and diastolic blood pressure values showed statistical significance. The average frequency of MS in patients with RA has been found as 22-44% in various studies [8, 10, 19]. This ratio was observed to be 28% in another study conducted in Turkey [20]. The rate of MS among our patients was 42% and this value is in line with the previous studies.

Although Akbal *et al*. have detected an increase in the frequency of MS in patients with RA, this increase did not show statistical significance. In their study, they have observed a higher prevalence of MS in the patients with lower functional status and detected a positive correlation between the stage of the disease and MS [20]. In our study, we have also found the prevalence of MS in the lower functional group as 69.2% and this value was significantly higher compared to the group with the higher functional status.

In a recent study, although no greater prevalence of MS was observed in patients with RA compared to the control group, the diastolic blood pressure was found to be significantly higher in the patients with RA in relation to the controls. This was also the case in our study. The lower frequency of MS observed in the same study was attributed to the all-female study subjects with a lower mean age than the previous studies [21].

It is a known fact that there is a relationship between the frequency of MS and the cardiovascular risk in patients with RA and this is greatly associated with morbidity and mortality [22]. In line with the literature data [8, 20], we have also detected significantly higher systolic and diastolic blood pressure values - components of MS - in patients with RA in comparison to the controls. Since the presence of MS is an important component of cardiovascular involvement and a higher prevalence of MS was observed in the patients with RA in our study, there may be a need in patients with RA for a follow-up in terms of MS and blood pressure values.

Karvounaris *et al*. have observed a significant correlation between the systolic blood pressure, which is a component of MS, and the DAS 28 score. We have also detected a similarly significant correlation between the systolic and diastolic blood pressure values and the DAS 28 scores in our study. It has also been observed in a recent study that the inflammation significantly increases the CVD risk markers (hypertension, intimal thickening, total cholesterol) in patients with active RA [23]. In the light of the data, we may conclude that the increase in the disease activity in patients with higher DAS 28 scores can lead to an increase the risk for hypertension.

Zonana *et al*. have detected an association between the frequency of MS, the short-term treatment with methotrexate and higher HAQ values in patients with RA. No relationship between the MS and ESR and RF were observed in the same study [24]. We have not observed any relationship between the HAQ, ESR, RF or the anti-CCP and MS in patients with RA in our study. The frequency of MS progresses in parallel to the patients’ age [25]. Also in our study, the mean age was significantly higher in the MS-positive patients with RA compared to the MS-negative patient group with RA.

### Limitations of the study

Any relationship between the medication and MS has not been investigated in this study. The medication used for the treatment of RA may take the disease activity under control and thus reduce the frequency of MS. Another limitation of the study was the small number of the patient and control groups compared to other studies.

### Conclusion

In conclusion, we have found the frequency of MS in patients with RA higher than the controls in our study. There was a significant relationship between the DAS 28 scores and the systolic and diastolic blood pressure values. In the patients with inferior functional status, the prevalence of MS was significantly higher than the patients with higher functional status. With an early detection of the MS and the choice of the appropriate treatment option in patients with RA, a decrease may be achieved in the risk for CDV, which causes significant numbers of morbidity and mortality. Therefore, studies with larger patient groups are needed to investigate the role of the MS in the development CVD in patients with RA in greater detail.

## Acknowledgement

We thank to all patients. This study was presented as a poster in the Excellence in Rheumatology Congress held in Istanbul, Turkey on 17-19 February 2011.
